# Supplementary material for: The roles of vision and antennal mechanoreception in hawkmoth flight control
Source: eLife. 2018 Dec 10;7:e37606. doi: 10.7554/eLife.37606 (PMC6303104; doi:10.7554/eLife.37606)
Supplement: Supplementary file 1. — The behaviour of each animal was classified into the following categories: no flight, flight (but no tracking of the flower), and tracking. Some moths were tested multiple times to collect the necessary tracking data, and thus have contributed multiple trials to this dataset. Statistical comparisons were performed using multinomial regression including the identity of individual moths as a random factor, to model the rates of one of the three behaviours as a function of antennal condition and lighting. As no significant interaction between antennal condition and light intensity was found, the fixed effects of the fitted model took the form: behavioural category (no flight, flight, tracking)~antennal condition+light intensity. All statistical results are expressed in relation to the probability of observing the no flight behaviour in the control condition in bright light. [file elife-37606-supp1.docx]

| **Conditions** | **Coefficient Estimate** | **t-value** | **p-value** |
| --- | --- | --- | --- |
| *flight* **ablate - control** | -1.27 | -1.53 | 0.126 |
| *tracking* **ablate - control** | -2.76 | -3.63 | <0.001 |
| *flight* **reatt - control** | -1.16 | -1.05 | 0.292 |
| *tracking* **reatt - control** | -0.83 | -0.88 | 0.380 |
| *flight* **dim - bright** | -1.01 | -1.93 | 0.054 |
| *tracking* **dim** **- bright** | -1.13 | -2.33 | 0.020 |
