# Supplementary material for: The roles of vision and antennal mechanoreception in hawkmoth flight control
Source: eLife. 2018 Dec 10;7:e37606. doi: 10.7554/eLife.37606 (PMC6303104; doi:10.7554/eLife.37606)
Supplement: Supplementary file 4. [file elife-37606-supp4.docx]

| **Treatment** | **Estimate** | **t-value** | **DF** | **p-value** |
| --- | --- | --- | --- | --- |
| **control** - **ablate** | -0.160 | -9.04 | 913 | <0.001 |
| **control** - **reatt** | -0.043 | -2.42 | 913 | 0.016 |
| **ablate** - **reatt** | 0.117 | 6.62 | 913 | <0.001 |
