# Supplementary material for: The roles of vision and antennal mechanoreception in hawkmoth flight control
Source: eLife. 2018 Dec 10;7:e37606. doi: 10.7554/eLife.37606 (PMC6303104; doi:10.7554/eLife.37606)
Supplement: Supplementary file 10. — A general linear model was constructed with antennal treatment and frequency (binned to the logarithmic scale) as factors: log(response)~antennal condition * frequency +1|individual. [file elife-37606-supp10.docx]

| **Treatment** | **Estimate** | **t-value** | **DF** | **p-value** |
| --- | --- | --- | --- | --- |
| **control** - **ablate** | -0.656 | -10.37 | 385 | <0.001 |
| **control** - **reatt** | -0.234 | -3.71 | 385 | 0.0002 |
| **ablate** - **reatt** | 0.421 | 6.67 | 385 | <0.001 |
