# Supplementary material for: The roles of vision and antennal mechanoreception in hawkmoth flight control
Source: eLife. 2018 Dec 10;7:e37606. doi: 10.7554/eLife.37606 (PMC6303104; doi:10.7554/eLife.37606)
Supplement: Supplementary file 11. [file elife-37606-supp11.docx]

| **Treatment** | **Estimate** | **t-value** | **DF** | **p-value** |
| --- | --- | --- | --- | --- |
| **control** - **ablate** | 0.184 | -6.36 | 462 | <0.001 |
| **control** - **reatt** | 0.028 | 0.98 | 462 | 0.328 |
| **ablate** - **reatt** | 0.156 | 5.38 | 462 | <0.001 |
