# Supplementary material for: The roles of vision and antennal mechanoreception in hawkmoth flight control
Source: eLife. 2018 Dec 10;7:e37606. doi: 10.7554/eLife.37606 (PMC6303104; doi:10.7554/eLife.37606)
Supplement: Supplementary file 13. — A Friedman test was performed, with a Tukey-Kramer post-hoc comparison correction for multiple comparisons. [file elife-37606-supp13.docx]

| **Treatment** | **Χ^2^** | **p-value** |
| --- | --- | --- |
| **overall** | 4.33 | 0.115 |
| **control** - **ablate** |  | 0.832 |
| **control** - **reatt** |  | 0.319 |
| **ablate** - **reatt** |  | 0.107 |
