# Supplementary material for: The roles of vision and antennal mechanoreception in hawkmoth flight control
Source: eLife. 2018 Dec 10;7:e37606. doi: 10.7554/eLife.37606 (PMC6303104; doi:10.7554/eLife.37606)
Supplement: Transparent reporting form [file elife-37606-transrepform.docx]

***eLife’s* transparent reporting form**

We encourage authors to provide detailed information *within their submission* to facilitate the interpretation and replication of experiments. Authors can upload supporting documentation to indicate the use of appropriate reporting guidelines for health-related research (see [EQUATOR Network](http://www.equator-network.org/%20)), life science research (see the [BioSharing Information Resource](https://biosharing.org/)), or the [ARRIVE guidelines](http://www.plosbiology.org/article/info:doi/10.1371/journal.pbio.1000412) for reporting work involving animal research. Where applicable, authors should refer to any relevant reporting standards documents in this form.

If you have any questions, please consult our Journal Policies and/or contact us: [editorial@elifesciences.org](mailto:editorial@elifesciences.org).

**Sample-size estimation**

- You should state whether an appropriate sample size was computed when the study was being designed
- You should state the statistical method of sample size computation and any required assumptions
- If no explicit power analysis was used, you should describe how you decided what sample (replicate) size (number) to use

Please outline where this information can be found within the submission (e.g., sections or figure legends), or explain why this information doesn’t apply to your submission:

We did not use power analysis to compute sample sizes. Sample size was determined based on experience from previous studies (Sponberg et al. (2015) Science 348: 1245–1248, Stöckl et al. (2017) Phil Trans R Soc Lond B, 372).

**Replicates**

- You should report how often each experiment was performed
- You should include a definition of biological versus technical replication
- The data obtained should be provided and sufficient information should be provided to indicate the number of independent biological and/or technical replicates
- If you encountered any outliers, you should describe how these were handled
- Criteria for exclusion/inclusion of data should be clearly stated
- High-throughput sequence data should be uploaded before submission, with a private link for reviewers provided (these are available from both GEO and ArrayExpress)

Please outline where this information can be found within the submission (e.g., sections or figure legends), or explain why this information doesn’t apply to your submission:

The data in *Table 1* includes all animals that were tested during the course of the moving flower experiments (in bright light: 38 control, 42 ablated, 20 re-attached, in dim light: 35 control, 66 ablated, 25 re-attached), also those that did not complete all conditions. Some animals were tested multiple times. The repeated measures and unbalanced design has been accounted for in the statistical analysis (see Table 1 and Table S1).

For the analysis of the moving and stationary flower experiments (Figs. 2-4, Figs. S2-3, Tables S2-S4), we only used animals that performed in all three antennal conditions, as well as in dim and bright light, to ensure a balanced experimental design with good control of individual effects.

We analyzed 6 individuals that performed in all antennal and light conditions in the stationary flower experiments, and 12 individuals that performed in all antennal and light conditions in the moving flower experiments (*Methods*, *Behavioural Experiments*).

**Statistical reporting**

- Statistical analysis methods should be described and justified
- Raw data should be presented in figures whenever informative to do so (typically when N per group is less than 10)
- For each experiment, you should identify the statistical tests used, exact values of N, definitions of center, methods of multiple test correction, and dispersion and precision measures (e.g., mean, median, SD, SEM, confidence intervals; and, for the major substantive results, a measure of effect size (e.g., Pearson's r, Cohen's d)
- Report exact p-values wherever possible alongside the summary statistics and 95% confidence intervals. These should be reported for all key questions and not only when the p-value is less than 0.05.

Please outline where this information can be found within the submission (e.g., sections or figure legends), or explain why this information doesn’t apply to your submission:

All our statistical analysis has been described (and referenced where applicable) in the Methods section (*Data analysis*) and in brief in the respective figure and table legends. Raw data is shown in Figs. 2-3 and S.2.1. The figure legends indicate the N values, statistical tests used, as well as population measures displayed, in addition to the information provided in the Methods section. All p-values have been reported, in addition to summary statistics, in supplementary tables (Tables S1-S4) relating to all data analysis.

(For large datasets, or papers with a very large number of statistical tests, you may upload a single table file with tests, Ns, etc., with reference to sections in the manuscript.)

**Group allocation**

- Indicate how samples were allocated into experimental groups (in the case of clinical studies, please specify allocation to treatment method); if randomization was used, please also state if restricted randomization was applied
- Indicate if masking was used during group allocation, data collection and/or data analysis

Please outline where this information can be found within the submission (e.g., sections or figure legends), or explain why this information doesn’t apply to your submission:

n.a.

**Additional data files (“source data”)**

- We encourage you to upload relevant additional data files, such as numerical data that are represented as a graph in a figure, or as a summary table
- Where provided, these should be in the most useful format, and they can be uploaded as “Source data” files linked to a main figure or table
- Include model definition files including the full list of parameters used
- Include code used for data analysis (e.g., R, MatLab)
- Avoid stating that data files are “available upon request”

Please indicate the figures or tables for which source data files have been provided:

All data generated or analysed during this study are included in the manuscript and supporting files. Source data files have been provided for Figures 2 and 3, as well as S2.1, S2.2 and S3.1.
